# Clinicopathologic Determinants of Overall Survival in Adrenocortical Carcinoma: A SEER-Based Population Study

**DOI:** 10.3390/cancers18071103

**Published:** 2026-03-28

**Authors:** Anıl Yıldız, Oguzcan Kınıkoğlu

**Affiliations:** 1 Department of Medical Oncology, Başakşehir Çam ve Sakura City Hospital, Başakşehir Neighborhood, G-434 Street, No: 2L, 34480 İstanbul, Türkiye; 2 Department of Medical Oncology, Kartal Dr. Lutfi Kirdar Education and Research Hospital, 34865 İstanbul, Türkiye; ogokinikoglu@yahoo.com

**Keywords:** adrenocortical carcinoma, SEER database, overall survival, prognostic factors, Kaplan–Meier analysis, cox regression, rare cancers, population-based study

## Abstract

**Background:** Adrenocortical carcinoma (ACC) is a rare and aggressive endocrine malignancy, for which population-level evidence regarding prognostic factors and survival conditions is limited. The available data mostly represent single-institution series, limiting their applicability. This study, therefore, assesses clinicopathological features and determines independent predictive variables of overall survival (OS) in patients with ACC using a population-based cohort. **Methods:** This retrospective observational cohort study used data from the Surveillance, Epidemiology, and End Results (SEER) Program between 2000 and 2022, initially identifying 1176 patients with ACC. Adult patients (≥18 years) with histologically confirmed ACC were identified using ICD-O-3 histology code 8370/3 and primary site code C74.0. Cases with zero-month survival, missing survival data, or identified only through autopsy or death certificate were excluded. To ensure dataset harmonization, patients with missing or indeterminate tumor grade and unknown stage were also excluded. After applying these inclusion and exclusion criteria, the final analytic cohort consisted of 267 patients. Data on demographic factors, stage of the disease, and treatment (surgery, chemotherapy, radiotherapy) were extracted. OS was evaluated using the Kaplan–Meier method, and independent prognostic factors were identified using Cox proportional hazards regression analysis. **Results:** The median OS was 54 months [95% confidence intervals (CI): 36–85]. The estimated 1-, 3-, and 5-year OS rates were 77%, 57%, and 48%, respectively. Survival differed significantly according to tumor grade, stage, and surgical treatment. In multivariable Cox regression analysis, increasing age [Hazard ratio (HR): 1.03, 95% CI: 1.02–1.04; *p* < 0.001], high tumor grade (HR: 2.21, 95% CI: 1.43–3.41; *p* < 0.001), and distant-stage disease (HR: 3.24, 95% CI: 1.95–5.38; *p* < 0.001) were independently associated with an increased risk of mortality, whereas surgical treatment was associated with improved survival (HR 0.53, 95% CI 0.30–0.93; *p* = 0.028). Chemotherapy and radiotherapy were not significantly associated with mortality. **Conclusions:** In this SEER-based cohort of patients with adrenocortical carcinoma, older age, high tumor grade, and distant-stage disease were independently associated with worse OS, whereas documented receipt of surgery was associated with longer OS. Treatment-related associations should be interpreted cautiously in view of the inherent limitations of registry-based data. Further prospective multicenter studies are needed to confirm these findings.

## Simple Summary

Adrenocortical carcinoma is a rare and aggressive cancer with limited population-level evidence regarding survival determinants. Using a large national cancer registry, we analyzed clinical characteristics and outcomes of patients diagnosed over a 22-year period. Older age, high tumor grade, and distant-stage disease were associated with worse overall survival, whereas receipt of surgery was associated with longer survival. Although different treatments were recorded, their apparent effect on survival could not be determined because registry data do not contain detailed clinical information such as surgical margins or specific drug regimens. These findings highlight the importance of early detection and careful interpretation of treatment outcomes in large databases. Future studies combining clinical and molecular information are needed to improve prognosis assessment and guide therapy.

## 1. Introduction

Adrenocortical carcinoma (ACC) is a rare and highly aggressive endocrine malignancy arising from the adrenal cortex, characterized by marked biological heterogeneity and poor prognosis [1]. Despite advances in oncologic diagnosis and treatment, ACC remains a major clinical challenge because of its low incidence, frequent presentation at advanced stages, and the limited availability of high-level evidence to guide management [2,3]. The reported annual incidence is approximately 0.5–2 cases per million, accounting for less than 0.2% of all malignant tumors [4]. Despite its rarity, ACC is associated with disproportionately high disease-specific mortality, underscoring its clinical importance [5]. Because of its low incidence, most available evidence originates from retrospective institutional series or relatively small multicenter cohorts, limiting the generalizability of prognostic and therapeutic findings [3,6].

ACC is a disease with a wide age distribution and follows a bimodal distribution in children and adults; however, in population-based studies, most cases are reported in adult-onset diseases [7]. A large percentage of those patients are also admitted to hospital with advanced-stage disease, which is due to the generally lethargic or non-specific clinical disease manifestation during the initial tumor progression [8]. ACC exhibits substantial heterogeneity in both clinical presentation and disease course [9]. Tumors may be hormonally functional or nonfunctional, and hormone excess can produce major systemic manifestations such as Cushing syndrome, virilization, feminization, hypertension, and metabolic disturbances [10,11]. Pathologically and clinically, ACC is associated with aggressive features including local invasion, early metastatic spread, and high recurrence risk [12]. The peritoneum, bones, lungs, and liver are common metastatic sites [13]. Prognosis is particularly poor in advanced disease, and stage at diagnosis has consistently emerged as one of the strongest determinants of survival [14,15].

Complete surgical resection remains the cornerstone of treatment and the only potentially curative option for localized ACC [16,17]. However, management becomes considerably more complex in locally advanced or metastatic disease, where patient selection, resectability, and the role of multimodal treatment remain matters of ongoing debate [18,19]. Systemic therapies, including mitotane and combination chemotherapy regimens, are widely used in advanced disease, yet survival benefits have been inconsistent across studies [20,21,22]. Radiotherapy is generally used for local control or palliation rather than as a clearly established survival-prolonging treatment [23,24]. These uncertainties reflect both the biological complexity of ACC and the limitations of the current evidence base.

Because ACC is rare, population-based registries offer an important opportunity to evaluate outcomes in larger and more representative cohorts. The Surveillance, Epidemiology, and End Results (SEER) program provides real-world demographic, clinicopathological, treatment, and survival data across multiple U.S. regions, making it particularly useful for the study of uncommon malignancies [25,26]. In addition to enabling larger cohorts, registry-based data may better reflect routine clinical practice and survival patterns than highly selected institutional series. Although prior registry-based studies have examined ACC outcomes, further contemporary analyses using large population-level datasets remain valuable for refining prognostic assessment and clarifying survival-associated factors [27]. Therefore, the present study aimed to evaluate clinicopathological characteristics and identify factors associated with overall survival (OS) in patients with ACC using a SEER-based cohort.

## 2. Materials and Methods

### 2.1. Data Source and Study Population

This retrospective observational cohort study used data from the SEER Program of the U.S. National Cancer Institute. Case extraction was performed using SEER*Stat software, version 9.0.42.2, from the database “Incidence—SEER Research Data, 17 Registries, Nov 2024 Sub (2000–2022)—Linked to County Attributes—Time Dependent (1990–2023) Income/Rurality, 1969–2023 Counties,” released in April 2025 based on the November 2024 submission.

Adult patients (≥18 years) diagnosed with ACC between 2000 and 2022 were identified using the International Classification of Diseases for Oncology, Third Edition (ICD-O-3) histology code 8370/3 and primary site code C74.0 (cortex of adrenal gland). To restrict the cohort to first and only primary ACC, cases were limited to those coded as “one primary only” in the SEER sequence number variable. Patients with zero-month survival or missing survival data were excluded. Cases identified only at autopsy or by death certificate were not retained because only patients with documented survival time and active follow-up were included. The detailed SEER*Stat session printout showing all case selection criteria and extracted variables is provided in Supplementary File S1.

**Figure 1:**
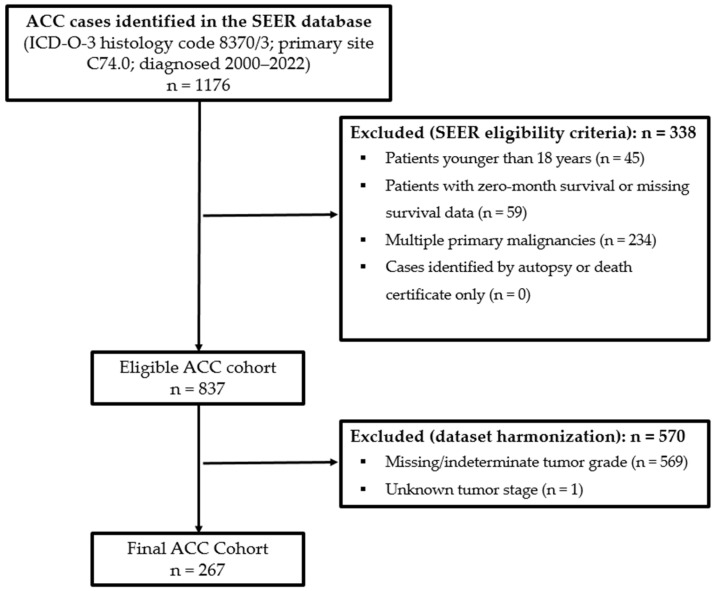
Patient selection.

After applying these initial eligibility criteria, 837 patients were identified. Tumor grade and stage variables were subsequently harmonized across diagnosis years using multiple SEER variables. During this process, 569 patients were excluded because tumor grade remained coded as blank, unknown, or indeterminate after harmonization, and one additional patient was excluded because tumor stage remained unknown. After these exclusions, all remaining patients had documented surgery status according to the RX Summ–Surg Prim Site (1998+) variable. Cases coded as No/Unknown for chemotherapy or radiotherapy were not excluded and were classified as having no documented receipt of treatment. The final analytic cohort consisted of 267 patients. The cohort derivation process is summarized in Figure 1, which illustrates the sequential application of inclusion and exclusion criteria.

Because the SEER database contains de-identified publicly available data, institutional review board approval and informed consent were not required.

### 2.2. Variables and Definitions

Outcome: OS was defined as the time in months from diagnosis to death from any cause or last follow-up. Follow-up time was defined as the interval from diagnosis to death or last contact, as recorded in the SEER database.

Demographic variables: Age at diagnosis was analyzed as a continuous variable, and sex was categorized as male or female.

Tumor grade: Tumor grade was harmonized across diagnosis years using two SEER variables: Grade Recode (thru 2017) and Grade Pathological (2018+). As the SEER dataset does not contain specific adrenal tumor parameters such as the Weiss score or Ki-67 proliferation index, these biomarkers could not be incorporated into the grading assessment. For cases diagnosed through 2017, grade information was obtained from Grade Recode (thru 2017), which includes the categories well differentiated (Grade I), moderately differentiated (Grade II), poorly differentiated (Grade III), and undifferentiated/anaplastic (Grade IV). For cases diagnosed in 2018 or later, grade information was obtained from Grade Pathological (2018+), which includes the codes L (low grade), and H (high grade). A unified tumor grade variable was constructed by merging these two variables and categorizing tumors as low grade or high grade. Low grade tumors were defined as Grade I–II in Grade Recode (thru 2017) or code L in Grade Pathological (2018+), whereas high grade tumors were defined as Grade III–IV in Grade Recode (thru 2017) or codes H in Grade Pathological (2018+). During the harmonization process, if one variable contained valid grade information while the other was coded as Blank(s), Unknown, or 9, the available valid value was used. Cases in which grade information remained Blank(s), Unknown, or 9 after this harmonization process were excluded from grade-related analyses.

Tumor stage: Tumor stage was harmonized across diagnosis years using SEER summary stage variables and categorized as localized, regional, or distant. Stage information was primarily obtained from SEER Historic Stage A (1973–2015) and Combined Summary Stage with Expanded Regional Codes, while Summary Stage 2000 (1998–2017) was also reviewed but contained only blank values in the extracted dataset and therefore did not contribute to stage classification. In SEER Historic Stage A, cases coded as Localized, Regional, and Distant were assigned directly to the corresponding study stage groups. In Combined Summary Stage with Expanded Regional Codes, Localized only was classified as localized; Regional lymph nodes involved only, Regional by direct extension only, and Regional by both direct extension and lymph node involvement were classified as regional; and Distant site(s)/node(s) involved was classified as distant. During the harmonization process, if one stage variable contained valid stage information while the other was coded as No/Unknown, Unknown/unstaged/unspecified/DCO, or Blank(s), the available valid value was used. Cases in which stage information remained unavailable after harmonization, including those coded as No/Unknown, Unknown/unstaged/unspecified/DCO, or Blank(s) without a valid corresponding value in the other stage variable, were excluded from the study.

Surgery for primary tumor: Surgery of the primary tumor was determined using the SEER variable RX Summ–Surg Prim Site (1998+). Cases with surgical procedure codes 20–90 were classified as having undergone surgery, whereas code 0 indicated no surgery.

Chemotherapy status was derived from the SEER variable Chemotherapy recode (yes, no/unk) and categorized as yes when coded as “Yes” and no when coded as “No/Unknown.”

Radiotherapy status was derived from the SEER variable Radiation recode and categorized as yes when coded as beam radiation or radioactive implants (including brachytherapy) and no when coded as none/unknown, recommended but unknown if administered, or refused.

Because the SEER database does not distinguish treatment intent, timing, or regimen, treatment variables were analyzed as receipt vs. no documented receipt.

### 2.3. Handling of Missing Data

Missing data were handled according to the structure and coding rules of each SEER variable. For tumor grade and tumor stage, harmonized variables were created across diagnosis years using multiple SEER fields, as described above. Cases with noninformative values (e.g., Blank(s), Unknown, 9, or equivalent unstaged/unspecified categories) that could not be resolved through harmonization were excluded from the analytic cohort. For treatment variables, the SEER database frequently groups “No” and “Unknown” categories. Consistent with prior registry-based survival studies, unknown treatment status was classified as no documented treatment. No imputation was performed. Available-case analysis was used for all variables.

### 2.4. Statistical Analysis

All statistical analyses were performed using IBM SPSS Statistics for Windows, version 20.0 (IBM Corp., Armonk, NY, USA). Descriptive statistics were summarized as mean ± standard deviation (SD) or median (interquartile range, IQR) for continuous variables, and as counts and percentages for categorical variables. OS was estimated using the Kaplan–Meier method and compared across groups using the log-rank test. Median OS with 95% confidence intervals (CIs) was reported. Cox proportional hazards regression was used to estimate hazard ratios (HRs) and 95% CIs. The proportional hazards assumption was assessed using log-minus-log survival plots and Schoenfeld residual inspection. Univariate Cox models were first fitted for each candidate covariate (age, sex, tumor stage, tumor grade, surgery, chemotherapy, radiotherapy). Variables with *p* < 0.10 in univariate analysis were considered for inclusion, while gender, chemotherapy, and radiotherapy variables were retained based on clinical relevance and their potential role as confounders in this observational registry-based cohort. Thus, the final multivariable model was specified on the basis of both statistical and clinical considerations, rather than by a purely automated variable selection procedure. The multivariable Cox model was fitted using forced entry (Enter) of the final covariate set. Variance inflation factors (VIFs) were used to assess multicollinearity, and a threshold of VIF > 5 was adopted to indicate multicollinearity [28]. Two-sided *p*-values < 0.05 were considered statistically significant.

## 3. Results

### 3.1. Baseline Characteristics

The final analytic cohort consisted of 267 patients with histologically confirmed ACC diagnosed between 2000 and 2022 in the SEER Research Data, 17 Registries. The majority of patients were female (60.3%), and the mean age at diagnosis was 54.0 ± 15.9 years. High-grade tumors were more common than low-grade tumors (61.8% vs. 38.2%). Regarding disease stage, 40.1% of patients had localized disease, 29.6% had regional disease, and 30.3% presented with distant metastasis. Most patients underwent surgery (92.1%). Chemotherapy and radiotherapy were administered in 41.9% and 24.0% of patients, respectively. The median follow-up time was 21 months (IQR: 9.0–43.0) (Table 1).

**Figure 2:**
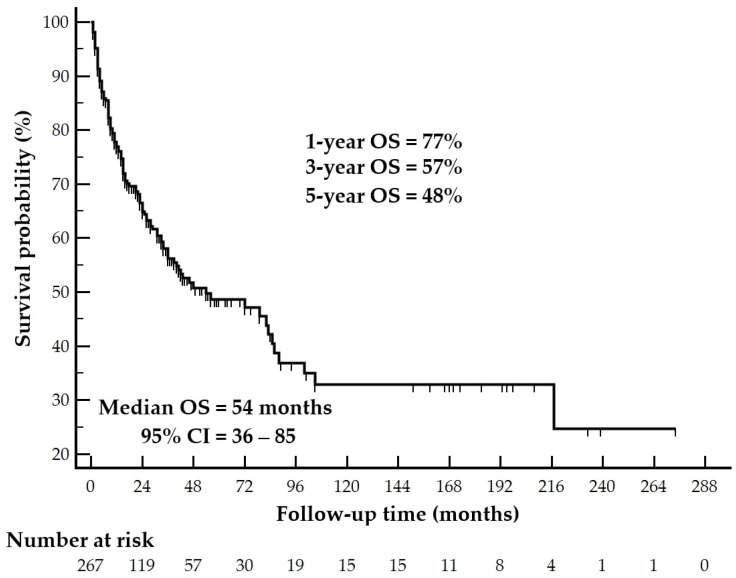
Overall survival of patients with adrenocortical carcinoma from the SEER database. Tick marks indicate censored observations, and the number of patients at risk at each time point is shown below the *x*-axis.

Among the 267 patients included in the SEER cohort, 149 were alive (55.8%) and 118 were deceased (44.2%) at the end of follow-up. The Kaplan–Meier analysis demonstrated that the median OS was 54 months (95% CI: 36–85). The estimated OS rates were 77% at 1 year, 57% at 3 years, and 48% at 5 years (Figure 2).

### 3.2. Factors Associated with Mortality

In univariate regression analysis, older age (HR: 1.03, 95% CI: 1.02–1.04; *p* < 0.001), high tumor grade (HR: 2.56, 95% CI: 1.67–3.93; *p* < 0.001), regional stage (HR: 1.68, 95% CI: 1.01–2.79; *p* = 0.044), and distant-stage disease (HR: 4.22, 95% CI: 2.72–6.56; *p* < 0.001) were associated with an increased risk of mortality. Surgical treatment was associated with a reduced mortality risk (HR: 0.21, 95% CI: 0.13–0.34; *p* = 0.001). In the multivariable model, increasing age (HR 1.03, 95% CI 1.02–1.04; *p* < 0.001), high tumor grade (HR: 2.21, 95% CI: 1.43–3.41; *p* < 0.001), and distant stage (HR: 3.24, 95% CI: 1.95–5.38; *p* < 0.001) remained independently associated with increased mortality risk, whereas surgical treatment was associated with a lower risk of mortality (HR: 0.53, 95% CI: 0.30–0.93; *p* = 0.028). Chemotherapy and radiotherapy were not significantly associated with mortality in either univariate or multivariable analyses. Multicollinearity was not identified among the variables included in the multivariable regression analysis (Table 2).

**Figure 3:**
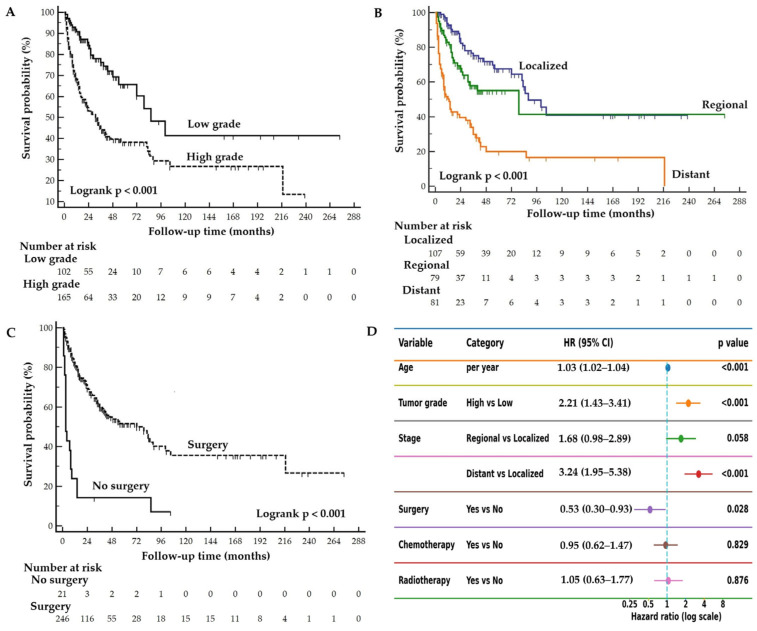
Overall survival stratified by tumor grade (**A**), tumor stage (**B**), and surgery (**C**) in adrenocortical carcinoma patients from the SEER database, and forest plot (**D**) illustrating the multivariable Cox regression model for mortality risk. Tick marks represent censored observations, and the number of patients at risk at each time point is displayed below the *x*-axis.

Kaplan–Meier survival analyses demonstrated significant differences in OS according to tumor grade, stage, and surgical treatment (Figure 3). Patients with high-grade tumors had significantly worse survival compared with those with low-grade tumors (log-rank *p* < 0.001) (Figure 3A). When stratified by SEER stage, survival outcomes progressively worsened from localized to regional and distant disease. Patients with distant stage ACC exhibited the poorest prognosis, whereas those with localized disease showed the most favorable survival outcomes (log-rank *p* < 0.001) (Figure 3B). Patients who underwent surgical resection had significantly higher survival probabilities compared with patients who did not undergo surgery (log-rank *p* < 0.001) (Figure 3C). The forest plot summarizing the multivariable Cox regression model is presented in Figure 3D.

Patients with low-grade tumors had longer median OS compared with those with high-grade tumors (86 vs. 31 months), with corresponding 1-, 3-, and 5-year survival rates of 92%, 77%, and 64% for low-grade tumors versus 69%, 46%, and 38% for high-grade tumors. Patients with localized disease showed the most favorable outcomes (median OS 88 months; 1-, 3-, and 5-year OS: 94%, 77%, and 68%), followed by those with regional disease (79 months; 83%, 57%, and 54%), whereas patients with distant disease had the poorest prognosis (13 months; 51%, 33%, and 19%). Patients who underwent surgery showed markedly longer observed survival than those who did not undergo surgery, with a median OS of 72 months versus 3 months, respectively, and corresponding 1-, 3-, and 5-year survival rates of 82%, 61%, and 51% versus 24%, 14%, and 14% (Table 3).

To further assess the robustness of mortality-related findings in the overall cohort (Table 2), subgroup Cox regression analyses were conducted based on tumor grade and disease stage (early-stage [localized + regional] and advanced-stage disease) (Table 3). Increasing age was independently associated with mortality, consistent with the overall population, except in the advanced-stage disease subgroup. Distant stage was independently associated with mortality compared with localized stage in both the low-grade and high-grade subgroups. In contrast, regional stage was not associated with mortality compared with localized stage, consistent with the findings in the overall cohort. Regression analysis could not be performed in the early-stage disease group because all patients underwent surgical treatment. In the advanced-stage disease group, surgery was associated with lower observed mortality in both univariate and multivariable analyses. However, surgery lost its statistical significance in the multivariable regression analysis in both the low- and high-grade groups. Across all subgroups, chemotherapy and radiotherapy were not significantly associated with mortality in either univariate or multivariable analyses (Table 4).

## 4. Discussion

In this study, based on the analysis of data from patients with ACC obtained from the SEER registry over a 22-year period, the median OS was 54 months, and survival differed markedly according to tumor grade, stage, and surgical treatment. In multivariable analysis, older age, high tumor grade, and distant-stage disease were independently associated with increased mortality, whereas surgical treatment was independently associated with improved survival. By contrast, chemotherapy and radiotherapy were not independently associated with OS after adjustment for available covariates. These findings offer valuable population-level data in an area where small institutional series are overly prominent and offer clinically relevant data about risk stratification and patient counseling.

The survival outcomes observed in the present study appear somewhat more favorable than those reported in several previous registry-based ACC series. Median OS reported in previous registry studies has been reported as between 17 and 30 months in unselected ACC populations, with much lower survival rates among patients who present with metastatic disease [29,30,31]. Similar data on 5-year survival has been described in large European registry and multicenter cohort studies, with survival rates ranging between 20 and 40 percent depending on cancer stage and patient characteristics [32]. In a SEER-based analysis by Wang et al., the median OS was 22 months, and age, treatment of the primary site, chemotherapy, and tumor stage were identified as prognostic factors, with stage exerting the strongest effect [33]. More recently, Chen et al. reported a median OS of 25 months and a 5-year OS rate of 31.5% in another SEER-based cohort [34]. In the National Cancer Database analysis by Tella et al., median OS was 3.21 years, and higher age, high tumor grade, stage IV disease, and lack of surgery were associated with worse outcomes [35]. The comparatively longer survival in our cohort may reflect the more selected analytic population after exclusion of cases with unresolved grade and stage information, as well as the high proportion of surgically treated patients. Of note, 570 of the 837 patients remaining after application of the initial eligibility criteria were excluded during grade/stage harmonization. This may have preferentially retained patients with more complete diagnostic work-up and clinicopathological documentation, while disproportionately excluding patients with more aggressive, rapidly progressive, or incompletely characterized disease. Therefore, the extensive exclusions may have introduced selection toward patients with more complete records and evaluable outcomes. Accordingly, the survival patterns identified here may reflect a more fully documented subset of ACC cases rather than the entire spectrum represented in SEER, and thus warrant cautious interpretation with respect to external validity.

Among all prognostic variables evaluated, disease stage remained one of the strongest determinants of outcome. Patients with distant-stage disease had substantially worse OS than those with localized disease, and Kaplan–Meier analyses showed a clear stage-dependent gradient in survival. This is consistent with prior population-based and registry studies showing that stage is the dominant prognostic factor in ACC [33,34,35,36]. In addition, high tumor grade emerged as an independent predictor of worse OS in the overall cohort, which is biologically plausible and concordant with prior literature indicating that higher-grade or more proliferative tumors have more aggressive clinical behavior [34,37]. Notably, in subgroup analyses, distant stage remained independently associated with mortality in both low-grade and high-grade tumors, whereas the effect of regional stage was attenuated, suggesting that metastatic spread carries prognostic weight beyond histologic differentiation alone.

Older age was also independently associated with worse OS in the overall cohort and remained associated with mortality in most subgroup analyses, except in advanced-stage disease. This finding is in line with prior large database studies in ACC, including both SEER and NCDB analyses, which have consistently identified age as an adverse prognostic factor [33,34,38,39]. The loss of statistical significance in the advanced-stage subgroup should not be interpreted as evidence that age is unimportant in metastatic disease; rather, it likely reflects the smaller sample size and the overwhelming prognostic impact of advanced tumor burden in that subgroup. Elderly patients may experience worse outcomes owing to numerous factors, including a decline in physiological reserve, increased burden of comorbid conditions, variance in tumor biology, and loss of tolerance to aggressive treatment. Regarding ACC in particular, certain research has indicated that younger patients might possess better tumor biology and chances of responding to surgical operation and multimodal therapy [38,40]. Therefore, age should play an important role in clinical decision-making, particularly when individualizing treatment strategies for elderly patients.

One of the most notable observational findings of this study was the association between surgery and longer OS. Surgical resection is widely regarded as the cornerstone of treatment and the only potentially curative option for localized ACC [3]. Our results were consistent with prior large registry analyses reporting longer OS among surgically treated patients, including the NCDB study by Tella et al. [35]. A SEER-based study covering 1973–2014 reported that 118 of 290 patients with metastatic ACC underwent primary tumor surgery, whereas 172 did not, and that surgery of the primary tumor was associated with improved OS [41]. The magnitude of the observed difference in our cohort, including a median OS of 72 months in the surgery group versus 3 months in the no-surgery group, must be interpreted with substantial caution. In routine clinical practice, patients selected for surgery generally have more favorable disease characteristics, including resectable tumors, lower metastatic burden, and better performance status, whereas patients who do not undergo surgery often have advanced disease, poor clinical condition, or other contraindications. Therefore, in a registry-based analysis such as ours, the observed association between surgery and survival likely reflects both treatment selection and underlying prognosis, and should not be interpreted as causal evidence of surgical efficacy. In subgroup analyses, surgery remained associated with longer observed OS even in patients with distant-stage disease. This finding is consistent with retrospective reports in metastatic ACC, in which longer OS has frequently been observed among patients undergoing primary tumor resection; however, those studies, like the present analysis, are highly susceptible to confounding by indication and selection bias and therefore do not establish a causal survival benefit [42]. At the same time, surgery could not be re-estimated in the early-stage subgroup because all such patients underwent surgery, which itself reflects current clinical practice in potentially resectable non-metastatic ACC. Although propensity score adjustment or matched analyses can reduce measured treatment-selection bias in some observational settings, their value in the present dataset would be limited by the marked imbalance between the surgery and no-surgery groups and the lack of key variables influencing surgical candidacy in SEER, such as resectability, performance status, metastatic burden, margin status, and comorbidity. Future studies using richer clinical datasets and more advanced causal-inference approaches are needed to better estimate the independent effect of surgery on survival in ACC.

In this registry-based cohort, we did not observe an association between chemotherapy or radiotherapy and OS after adjustment. However, this finding is highly susceptible to confounding by indication, incomplete treatment capture, and misclassification. These findings should be interpreted with caution and should not be taken as evidence of lack of clinical benefit. In advanced ACC, mitotane-based therapy and combination chemotherapy remain standard components of treatment, particularly following the FIRM-ACT trial, but their impact on long-term survival is heterogeneous [43,44,45]. Similarly, the role of radiotherapy appears to be context-dependent. A SEER-based study by Thomas and Tward suggested that selected patients with stage III, node-negative disease may derive survival benefit from adjuvant radiotherapy [46], while studies in advanced ACC have generally supported radiotherapy more for local control or palliation than for a clear OS effect [24,47,48]. Accordingly, the non-significant estimates in our models should be interpreted as inconclusive rather than negative, particularly because the SEER database does not capture surgical margin status, mitotane use, chemotherapy regimen details, treatment sequencing, dose intensity, recurrence patterns, or performance status. Treatment variables in this setting therefore reflect treatment allocation as much as treatment efficacy and remain susceptible to residual confounding and confounding by indication.

From a clinical standpoint, the results of this study have several important implications for practicing oncologists, endocrinologists, and surgeons involved in the management of ACC. First, the strong prognostic impact of disease stage emphasizes the need for thorough staging evaluation at diagnosis and careful consideration of disease extent when counseling patients regarding prognosis. Second, the identification of age as an independent prognostic factor suggests that patient-related characteristics, in addition to tumor features, play a critical role in outcomes and should be integrated into risk stratification models. Third, the limited independent impact of current treatment modalities on survival underscores the importance of multidisciplinary management and referral to specialized centers with expertise in adrenal tumors, where patients may have access to clinical trials and emerging therapies. Taken together, these findings support a personalized, evidence-informed approach to ACC management that incorporates both disease-specific and patient-specific factors.

This study has several notable strengths. First, it uses a contemporary population-based SEER cohort, allowing evaluation of a relatively large sample for a rare malignancy and reducing referral bias compared with single-center series. Second, stage and tumor grade were explicitly harmonized across diagnosis years, improving the internal consistency of the analytic dataset. Third, survival was assessed using standardized Kaplan–Meier and Cox regression methods, and additional subgroup analyses were performed to examine whether the associations observed in the overall cohort remained directionally consistent across clinically relevant strata. However, due to the limited number of patients and events in certain subgroups, these analyses may have lacked sufficient statistical power and generated unstable effect estimates. Therefore, caution is warranted when interpreting these results. Together, these features enhance the generalizability, methodological transparency, and clinical relevance of the present findings.

### Study Limitations

This study has several limitations that should be considered when interpreting the findings. First, its retrospective observational registry-based design is inherently subject to selection bias, residual confounding, and unmeasured confounding. In addition, the analytic cohort was substantially reduced during variable harmonization: of the 837 patients remaining after application of the initial eligibility criteria, 570 were excluded because grade or stage could not be reliably harmonized across diagnosis years, including 569 with unresolved tumor grade and 1 with unknown stage. Although this approach improved internal consistency for grade- and stage-based analyses, it may have reduced external validity by preferentially retaining patients with more complete diagnostic and pathological documentation. Because missing or indeterminate registry data may be nonrandom, patients with more aggressive disease, limited work-up, incomplete documentation, or rapid clinical deterioration may have been disproportionately excluded. Therefore, the final cohort may not fully represent the broader SEER ACC population, and the observed survival outcomes may be somewhat more favorable than would be expected in an unselected cohort. Accordingly, our findings are most applicable to patients with more completely characterized ACC in SEER rather than to the entire unselected registry population. Second, the SEER database lacks several clinical, pathological, and molecular variables that are highly relevant in adrenocortical carcinoma. Important factors such as hormonal activity, Ki-67 index, Weiss score, margin status (R0 vs. R1/R2), recurrence patterns, performance status, comorbidity burden, and detailed systemic treatment data (including mitotane use, chemotherapy regimens, dose intensity, and treatment duration) are not available. The absence of these variables limits more refined risk adjustment and may contribute to residual confounding, particularly in the interpretation of treatment-related associations, as treatment allocation in clinical practice is closely linked to factors such as tumor biology, resectability, and patient performance status. This limited clinical granularity should be explicitly considered when interpreting both the prognostic estimates and the treatment-related associations reported in this study. Third, although the use of a large population-based dataset improves representativeness, it does not permit causal inference, particularly with respect to treatment effects. Accordingly, treatment-related results should be interpreted as observational associations rather than evidence of treatment efficacy. SEER treatment variables primarily reflect recorded receipt of first-course therapy rather than treatment intent, sequencing, completeness, or quality. These variables may also be affected by underreporting or misclassification. More importantly, patients who underwent surgery, chemotherapy, or radiotherapy are unlikely to be directly comparable with those who did not receive these treatments, because treatment allocation in routine practice is strongly influenced by disease extent, performance status, comorbidities, operability, and access to specialized care. This issue is particularly relevant for surgery, given the very large observed survival difference between surgically and non-surgically managed patients in our cohort. As a result, the observed treatment estimates are highly vulnerable to confounding by indication and selection bias, and the association between surgery and longer survival should not be interpreted as evidence of causal treatment efficacy. Although methods such as propensity score adjustment or matched analyses may reduce measured treatment-selection bias, their usefulness in the present dataset would still be limited by the marked imbalance between treatment groups and the absence of key clinical variables determining treatment selection. Therefore, the absence of an independent association between chemotherapy/radiotherapy and OS, as well as the favorable association observed for surgery, should be interpreted cautiously and as observational rather than causal findings. Fourth, although SEER provides robust OS data, it does not adequately capture other clinically relevant endpoints such as recurrence-free survival, progression-free survival, treatment response, patterns of relapse, or detailed cause-specific disease course. This limits a more nuanced evaluation of outcome trajectories in ACC. Moreover, the median follow-up duration in the selected cohort was relatively short, which may have reduced the ability to fully capture late mortality events and longer-term survival patterns in this biologically heterogeneous malignancy. Therefore, long-term outcome estimates should be interpreted cautiously.

Finally, the subgroup analyses were exploratory. Several subgroup models were based on relatively small numbers of patients and events, which may have limited statistical power and reduced estimate stability. Moreover, because multiple subgroup analyses were conducted without formal correction for multiple comparisons, the possibility of type I error cannot be excluded. Future studies using larger and more comprehensively characterized cohorts, with more balanced subgroup sizes and richer clinical detail, are needed to confirm these findings and to better assess the consistency of prognostic associations across clinically relevant strata. External validation in independent datasets will also be important to determine the robustness and generalizability of the present results.

## 5. Conclusions

In this population-based study, our findings reinforce that ACC remains a biologically aggressive malignancy in which patient age, tumor grade, and especially metastatic stage are major determinants of survival. They also support the continued central role of surgery in appropriately selected patients, while highlighting the difficulty of drawing firm conclusions regarding chemotherapy and radiotherapy from registry-level data alone. However, these findings should be interpreted in light of substantial case exclusion during harmonization, limited clinical detail, and the relatively short follow-up duration. Future studies integrating detailed pathological, clinical, and treatment-specific variables will be necessary to refine prognostic stratification and better define treatment benefit in ACC.

## Figures and Tables

**Table 1:** Baseline demographic and clinicopathological features of patients with adrenocortical carcinoma.

Variables	All Population n = 267
Gender, n (%)	
Female	161 (60.3)
Male	106 (39.7)
Age of diagnosis, years	54.0 ± 15.9
Tumor grade, n (%)	
Low	102 (38.2)
High	165 (61.8)
Stage, n (%)	
Localized	107 (40.1)
Regional	79 (29.6)
Distant	81 (30.3)
Surgery, n (%)	
No	21 (7.9)
Yes	246 (92.1)
Chemotherapy, n (%)	
No	155 (58.1)
Yes	112 (41.9)
Radiotherapy, n (%)	
No	203 (76.0)
Yes	64 (24.0)
Overall survival, months ^a^	54 (36–85)
Follow-up time, months	21.0 (9.0–43.0)

Data are mean ± standard deviation or median (IQR), or number (%). ^a^ Overall survival was presented as the median (95% CI).

**Table 2:** The relationship between demographic, clinicopathological, and treatment-related parameters and mortality.

Variables	Survival		Univariate Analysis		Multivariable Analysis		VIF
	Alive n = 149	Deceased n = 118	HR (95% CI)	*p*	HR (95% CI)	*p*	
Gender, n (%)							1.02
Female	90 (60.4)	71 (60.2)	ref		ref		
Male	59 (39.6)	47 (39.8)	1.09 (0.76–1.58)	0.633	1.01 (0.70–1.48)	0.942	
Age, years	50.0 ± 15.5	59.0 ± 15.0	1.03 (1.02–1.04)	<0.001 *	1.03 (1.02–1.04)	<0.001 *	1.05
Tumor grade, n (%)							1.09
Low	75 (50.3)	27 (22.9)	ref		ref		
High	74 (49.7)	91 (77.1)	2.56 (1.67–3.93)	<0.001 *	2.21 (1.43–3.41)	<0.001 *	
Stage, n (%)							1.32
Localized	76 (51.0)	31 (26.3)	ref		ref		
Regional	49 (32.9)	30 (25.4)	1.68 (1.01–2.79)	0.044 *	1.68 (0.98–2.89)	0.058	
Distant	24 (16.1)	57 (48.3)	4.22 (2.72–6.56)	<0.001 *	3.24 (1.95–5.38)	<0.001 *	
Surgery, n (%)							1.20
No	2 (1.3)	19 (16.1)	ref		ref		
Yes	147 (98.7)	99 (83.9)	0.21 (0.13–0.34)	0.001 *	0.53 (0.30–0.93)	0.028 *	
Chemotherapy, n (%)							1.16
No	88 (59.1)	67 (56.8)	ref		ref		
Yes	61 (40.9)	51 (43.2)	1.05 (0.73–1.51)	0.798	0.95 (0.62–1.47)	0.829	
Radiotherapy, n (%)							1.26
No	111 (74.5)	92 (78.0)	ref		ref		
Yes	38 (25.5)	26 (22.0)	1.05 (0.67–1.63)	0.841	0.96 (0.59–1.57)	0.876	
					−2 Log Likelihood = 1079		

Data are mean ± standard deviation or median (IQR), or number (%). * *p* < 0.05 indicates statistical significance. All VIF values < 5, indicating absence of significant multicollinearity. Abbreviations: CI, confidence intervals; HR, hazard ratio; ref, reference group; VIF, variance inflation factors.

**Table 3:** Median overall survival and survival rates by tumor grade, stage, and surgery.

Variable	OS (95% CI)	Survival Rates		
		1-Years	3-Years	5-Years
Tumor grade, n (%)				
Low	86 (57–114)	92%	77%	64%
High	31 (21–40)	69%	46%	38%
Stage, n (%)				
Localized	88 (66–109)	94%	77%	68%
Regional	79 (7–150)	83%	57%	54%
Distant	13 (7–18)	51%	33%	19%
Surgery, n (%)				
No	3 (2–4)	24%	14%	14%
Yes	72 (43–100)	82%	61%	51%

Data are presented as median overall survival (OS) in months with 95% confidence intervals (CI), and corresponding 1-, 3-, and 5-year overall survival rates (%). OS was estimated using the Kaplan–Meier method.

**Table 4:** Subgroup Cox regression analyses for mortality.

Subgroup	N	Events	Variables	Univariate Analysis		Multivariable Analysis	
				HR (95% CI)	*p*	HR (95% CI)	*p*
Early-stage disease (localized + regional)	186	61	Age (per year)	1.04 (1.03–1.06)	<0.001 *	1.05 (1.03–1.06)	<0.001 *
			Gender (Male vs. Female)	1.02 (0.60–1.72)	0.951	0.89 (0.52–1.52)	0.669
			Grade (High vs. Low)	2.63 (1.47–4.71)	0.001 *	2.49 (1.37–4.51)	0.003 *
			Surgery (Yes vs. No)	NE	—	NE	—
			Chemotherapy (Yes vs. No)	1.06 (0.63–1.79)	0.819	1.23 (0.66–2.28)	0.515
			Radiotherapy (Yes vs. No)	1.40 (0.77–2.54)	0.275	1.14 (0.57–2.30)	0.711
Advanced-stage disease (Distant stage)	81	57	Age (per year)	1.01 (0.99–1.03)	0.154	1.01 (0.99–1.03)	0.245
			Gender (Male vs. Female)	1.05 (0.62–1.78)	0.868	1.15 (0.67–1.97)	0.620
			Grade (High vs. Low)	1.78 (0.93–3.40)	0.082	1.74 (0.89–3.39)	0.107
			Surgery (Yes vs. No)	0.44 (0.25–0.76)	0.004 *	0.50 (0.28–0.90)	0.021 *
			Chemotherapy (Yes vs. No)	0.68 (0.40–1.15)	0.147	0.83 (0.46–1.47)	0.512
			Radiotherapy (Yes vs. No)	0.76 (0.39–1.48)	0.423	0.95 (0.47–1.90)	0.874
Low-grade tumors	102	27	Age (per year)	1.04 (1.01–1.07)	0.003 *	1.05 (1.01–1.08)	0.006 *
			Gender (Male vs. Female)	0.83 (0.37–1.86)	0.658	0.94 (0.40–2.20)	0.883
			Stage (Regional vs. Localized)	1.52 (0.54–4.27)	0.431	1.45 (0.50–4.18)	0.489
			Stage (Distant vs. Localized)	5.26 (2.18–12.72)	<0.001 *	4.38 (1.57–12.25)	0.005 *
			Surgery (Yes vs. No)	0.23 (0.07–0.79)	0.020 *	1.30 (0.25–6.71)	0.752
			Chemotherapy (Yes vs. No)	1.96 (0.91–4.21)	0.085	1.74 (0.68–4.45)	0.247
			Radiotherapy (Yes vs. No)	1.52 (0.59–3.88)	0.384	0.86 (0.29–2.59)	0.792
High-grade tumors	165	91	Age (per year)	1.03 (1.01–1.04)	<0.001 *	1.03 (1.01–1.04)	0.001 *
			Gender (Male vs. Female)	1.17 (0.77–1.78)	0.465	1.02 (0.66–1.56)	0.935
			Stage (Regional vs. Localized)	1.46 (0.81–2.62)	0.209	1.71 (0.91–3.24)	0.096
			Stage (Distant vs. Localized)	3.32 (1.98–5.57)	<0.001 *	3.15 (1.72–5.77)	<0.001 *
			Surgery (Yes vs. No)	0.25 (0.14–0.43)	<0.001 *	0.55 (0.28–1.05)	0.070
			Chemotherapy (Yes vs. No)	0.75 (0.49–1.13)	0.170	0.78 (0.46–1.30)	0.334
			Radiotherapy (Yes vs. No)	0.90 (0.55–1.49)	0.681	0.99 (0.57–1.74)	0.981

The reference groups were females for sex, low-grade for tumor grade, localized stage for disease stage, and the “No” group for surgery, chemotherapy, and radiotherapy. * *p* < 0.05 indicates statistical significance. Abbreviations: CI, confidence intervals; HR, hazard ratio; NE, not estimable.

## Data Availability

The data used in this study were obtained from the Surveillance, Epidemiology, and End Results (SEER) Program of the U.S. National Cancer Institute. Case extraction was performed using SEER*Stat software version 9.0.42.2 from the database “Incidence—SEER Research Data, 17 Registries, November 2024 Sub (2000–2022), released April 2025.” These registry data are publicly available and can be accessed through the SEER program upon request and approval (https://seer.cancer.gov/, access date: 20 January 2026).

## Supplementary Materials

The following supporting information can be downloaded at: https://www.mdpi.com/article/10.3390/cancers18071103/s1, Supplementary File S1: Summary of case listing.
