# Supplementary material for: Clinicopathologic Determinants of Overall Survival in Adrenocortical Carcinoma: A SEER-Based Population Study
Source: Cancers (Basel). 2026 Mar 28;18(7):1103. doi: 10.3390/cancers18071103 (PMC13072178; doi:10.3390/cancers18071103)
Supplement: Supplementary file 1 [file cancers-18-01103-s001.zip › cancers-4148927-supplementary.pdf]

Session Type: Case Listing

Suggested Citation

Software: Surveillance Research Program, National Cancer Institute SEER*Stat software (www.seer.cancer.gov/seerstat) version 9.0.42.2.
Data: Surveillance, Epidemiology, and End Results (SEER) Program (www.seer.cancer.gov) SEER*Stat Database: Incidence - SEER Research Data, 17 Registries, Nov 2024 Sub (2000-2022) - Linked To County Attributes - Time Dependent (1990-2023) Income/Rurality, 1969-2023 Counties, National Cancer Institute, DCCPS, Surveillance Research Program, released April 2025, based on the November 2024 submission.

Data

Database: Incidence - SEER Research Data, 17 Registries, Nov 2024 Sub (2000-2022) - Linked To County Attributes - Time Dependent (1990-2023) Income/Rurality, 1969-2023 Counties
Database ID: 2536
Link Database ID: 2593
Use Summary Files: No

Selection

Select Only: Known Age
Case: {Site and Morphology.ICD-O-3 Hist/behav, malignant} = '8370/3: Adrenal cortical carcinoma'
AND {Site and Morphology.Primary Site - labeled} = 'C74.0-Cortex of adrenal gland'
AND {Race, Sex, Year Dx.Year of diagnosis} = '2000-2022','2000','2001','2002','2003','2004','2005','2006','2007','2008','2009','2010','2011','2012','2013','2014','2015','2016','2017','2018','2019','2020','2021','2022'
AND {Multiple Primary Fields.Sequence number} = 'One primary only'
AND {Race and Age (case data only).Age recode with single ages and 90+} = '18 years','19 years','20 years','21 years','22 years','23 years','24 years','25 years','26 years','27 years','28 years','29 years','30 years','31 years','32 years','33 years','34 years','35 years','36 years','37 years','38 years','39 years','40 years','41 years','42 years','43 years','44 years','45 years','46 years','47 years','48 years','49 years','50 years','51 years','52 years','53 years','54 years','55 years','56 years','57 years','58 years','59 years','60 years','61 years','62 years','63 years','64 years','65 years','66 years','67 years','68 years','69 years','70 years','71 years','72 years','73 years','74 years','75 years','76 years','77 years','78 years','79 years','80 years','81 years','82 years','83 years','84 years','85 years','86 years','87 years','88 years','89 years','90+ years'
AND {Stage - Summary/Historic.SEER historic stage A (1973-2015)} = 'In situ','Localized','Regional','Distant','Localized/regional (Prostate cases)','Unstaged','Blank(s)'
AND {Stage - Summary/Historic.Summary stage 2000 (1998-2017)} = 'In situ','Localized','Regional','Distant','N/A','Unknown/unstaged','Blank(s)'
AND {Stage - Summary/Historic.Combined Summary Stage with Expanded Regional Codes (2004+)} = 'In situ','Localized only','Regional by direct extension only','Regional lymph nodes involved only','Regional by both direct extension and lymph node involvement','Distant site(s)/node(s) involved','Not applicable/Benign/Borderline','Unknown/unstaged/unspecified/DCO','Blank(s)'
AND {Site and Morphology.Grade Recode (thru 2017)} = 'Well differentiated; Grade I','Moderately differentiated; Grade II','Poorly differentiated; Grade III','Undifferentiated; anaplastic; Grade IV','T-cell','B-cell; pre-B; B-precursor','Null cell; non T-non B','NK cell; natural killer cell (1995+)','Unknown','Blank(s)'
AND {Site and Morphology.Grade Pathological (2018+)} = '1','2','3','4','5','8','9','A','B','C','D','E','L','H','M','S','Blank(s)'
AND {Therapy.RX Summ--Surg Prim Site (1998+)} = 0-99
AND {Therapy.Radiation recode} = 'None/Unknown','Beam radiation','Radioactive implants (includes brachytherapy) (1988+)','Combination of beam with implants or isotopes','Radiation, NOS method or source not specified','Refused (1988+)','Recommended, unknown if administered'
AND {Therapy.Chemotherapy recode (yes, no/unk)} = 'No/Unknown','Yes'
AND {Cause of Death (COD) and Follow-up.Survival months flag} = 'Complete dates are available and there are 0 days of survival','Complete dates are available and there are more than 0 days of survival','Incomplete dates are available and there could be zero days of follow-up','Incomplete dates are available and there cannot be zero days of follow-up','Not calculated because a Death Certificate Only or Autopsy Only case'
AND {Cause of Death (COD) and Follow-up.Survival months} = 1-275
AND {Cause of Death (COD) and Follow-up.Vital status recode (study cutoff used)} = 'Alive','Dead'
AND {Race, Sex, Year Dx.Sex} = ' Male',' Female'

10,334,116

Number Selected	Number Excluded	Statement
----------	----------	--
		User Selection (Case)
2,154	10,331,962	{ICD-O-3 Hist/behav, malignant} = '8370/3: Adrenal cortical carcinoma'
1,176	978	{Primary Site - labeled} = 'C74.0-Cortex of adrenal gland'
1,176	0	{Year of diagnosis} = '2000-2022','2000','2001','2002','2003','2004','2005','2006','2007', '2008','2009','2010','2011','2012','2013','2014','2015','2016','2017','2018','2019','2020', '2021','2022'
942	234	{Sequence number} = 'One primary only'
897	45	{Age recode with single ages and 90+} = '18 years','19 years','20 years','21 years','22 years','23 years','24 years','25 years','26 years','27 years','28 years','29 years','30 years','31 years','32 years','33 years','34 years','35 years','36 years','37 years','38 ... (see Session Printout)
897	0	{SEER historic stage A (1973-2015)} = 'In situ','Localized','Regional','Distant', 'Localized/regional (Prostate cases)','Unstaged','Blank(s)'
897	0	{Summary stage 2000 (1998-2017)} = 'In situ','Localized','Regional','Distant','N/A', 'Unknown/unstaged','Blank(s)'
897	0	{Combined Summary Stage with Expanded Regional Codes (2004+)} = 'In situ','Localized only','Regional by direct extension only','Regional lymph nodes involved only','Regional by both direct extension and lymph node involvement','Distant site(s)/node(s) involved', ... (see Session Printout)
897	0	{Grade Recode (thru 2017)} = 'Well differentiated; Grade I','Moderately differentiated; Grade II','Poorly differentiated; Grade III','Undifferentiated; anaplastic; Grade IV', 'T-cell','B-cell; pre-B; B-precursor','Null cell; non T-non B','NK cell; natural killer ... (see Session Printout)
897	0	{Grade Pathological (2018+)} = '1','2','3','4','5','8','9','A','B','C','D','E','L','H', 'M','S','Blank(s)'
897	0	{RX Summ--Surg Prim Site (1998+)} = 0-99
896	1	{Radiation recode} = 'None/Unknown','Beam radiation','Radioactive implants (includes brachytherapy) (1988+)','Combination of beam with implants or isotopes','Radiation, NOS method or source not specified','Refused (1988+)','Recommended, unknown if administered'
896	0	{Chemotherapy recode (yes, no/unk)} = 'No/Unknown','Yes'
896	0	{Survival months flag} = 'Complete dates are available and there are 0 days of survival', 'Complete dates are available and there are more than 0 days of survival','Incomplete dates are available and there could be zero days of follow-up','Incomplete dates are ... (see Session Printout)
837	59	{Survival months} = 1-275
837	0	{Vital status recode (study cutoff used)} = 'Alive','Dead'
837	0	{Sex} = ' Male',' Female'
		Standard Exclusions
837	0	Select only known age
